# Patient-Led Research Collaborative: embedding patients in the Long COVID narrative

**DOI:** 10.1097/PR9.0000000000000913

**Published:** 2021-04-13

**Authors:** Lisa McCorkell, Gina S. Assaf, Hannah E. Davis, Hannah Wei, Athena Akrami

**Affiliations:** a Patient-Led Research Collaborative, Washington DC, USA,; b Sainsbury Wellcome Centre, University College London, London, United Kingdom

**Keywords:** Long COVID, COVID-19, Patient-led research, Patient-centric, Long haulers, Post-acute sequelae of SARS-CoV-2 infection, Post COVID-19 syndrome, Patient-centered

## Abstract

A large subset of patients with coronavirus disease 2019 (COVID-19) are experiencing symptoms well beyond the claimed 2-week recovery period for mild cases. These long-term sequelae have come to be known as Long COVID. Originating out of a dedicated online support group, a team of patients formed the Patient-Led Research Collaborative and conducted the first research on Long COVID experience and symptoms. This article discusses the history and value of patient-centric and patient-led research; the formation of Patient-Led Research Collaborative as well as key findings to date; and calls for the following: the acknowledgement of Long COVID as an illness, an accurate estimate of the prevalence of Long COVID, publicly available basic symptom management, care, and research to not be limited to those with positive polymerase chain reaction and antibody tests, and aggressive research and investigation into the pathophysiology of symptoms.

## Abstract 2

This article discusses the value of patient-led research, the formation of Patient-Led Research for COVID-19, and how to improve Long COVID care and research.

## 1. Introduction

During the first few months of the coronavirus disease 2019 (COVID-19) pandemic, a false narrative was presented to the public: most infected people would recover within 2 weeks.^24^ However, a large subset of patients with COVID-19, many of them young and previously healthy, continued to experience symptoms well past 2 weeks.^4–7,11,12,34^ These patients have what is now referred to as Long COVID,^28^ which we define as being symptomatic from COVID-19 for more than 28 days, with many experiencing debilitating symptoms for months.^10^

On April 13, 2020, Fiona Lowenstein published an op-ed in the *New York Times* about Long COVID recoveries, which brought thousands of people to the support group her organization created for patients experiencing these prolonged symptoms, called the Body Politic COVID-19 Support Group.^21^ Each of the authors of this article joined, looking for support and for answers. Out of the support group, a research team was born with the initial goal to gather and document people's various experiences with this illness. By being patient driven and patient centric, the Patient-Led Research Collaborative (PLRC) COVID-19 team has been able to better understand patient concerns, document what patients are experiencing, and create surveys that reflect these concerns and experiences.

This article discusses the importance of patient-led and patient-involved research, the formation of the PLRC, and the gaps that still exist in the provision of care in patients with Long COVID.

## 2. Patient-led research

In conventional medical research, doctors and researchers decide what outcomes matter and what hypotheses should be tested. When patients with the illness being studied are not included in these decisions, there is often a discrepancy between the research that patients want and the research that actually happens.^14,15,29^ Several medical establishments and leading publishers advocate for research that involves patients in decision making. For example, the *British Medical Journal* has set up a partnership with patients and the public to encourage this type of work,^37^ and the Patient-Centered Outcomes Research Institute (PCORI) promotes research guided by patients in the United States.^27^

A step beyond this work is patient-led research, in which patients are not only involved, but actually lead the research. Although some critics say that patients may not have the education needed to do their own research,^1^ there is no degree that can give you the lived experience of an illness or the collective knowledge of an online community.^36^ People experiencing the illness are best able to identify the questions to ask and issues to investigate that matter to them and also to design effective solutions based on their intimate familiarity of the illness. Too often conventional research tests hypotheses that do not address the urgent needs of patients and takes too long for many patients to benefit from. Patient-led research, on the other hand, attempts to answer the questions that will most help patients while being able to release results quickly and publicly. After all, the point of research is to provide access to trusted information and to contribute to our shared knowledge and understanding of an illness while finding treatments that improve patients' quality of life. Patient-led research does this effectively and efficiently.

This type of research does come with its own unique set of challenges. First, patients are still patients. It is important for them to prioritize their health above the research, even when that may result in slower progress than what an ableist world demands. Second, being able to sustain a research group requires both an organizational structure and funding. Patients who are self-organized and outside of the conventional research system do not begin with this structure in place. This also leads to the next challenge, which is that there is an initial limit to the type of research patients can perform. For example, without partnerships, most patients do not have all of the skills or infrastructure access needed to complete a randomized control trial, which is essential in preventing biases in the collection and interpretation of data.

Despite these challenges, there are countless examples of patients who have led their own research to date, many stemming from online health communities.^17^ Patients with amyotrophic lateral sclerosis, Parkinson disease, and diabetes have paved the way in conducting impactful research for and with their communities.^41^ Their findings have made a large impact on the communities they are part of and highlight the critical importance of patient-led research.

## 3. Patient-Led Research Collaborative

### 3.1. Creation of the research group

The Body Politic COVID-19 Support Group^31^ initially started on a messaging platform called WhatsApp but quickly moved to a larger platform called Slack as the number of members increased beyond WhatsApp's capacity. People were eager to share their story and see if their symptoms aligned with others', particularly because there was little public health, medical, or media attention on this extended version of the illness. This type of support group is not a new phenomenon. When doctors are at a loss for answers or provide diagnoses of anxiety without also providing treatment, patients of unresearched or ignored illnesses often seek out others who are experiencing their same symptoms—for validation, for advice, and for treatment.^2,13,16,18,27^ The Body Politic Support Group's reach expanded particularly quickly because the nature of COVID led to a large number of people having similar experiences at the same time.

The Slack group is organized into dedicated channels for different topics and symptoms. There are more than 60 channels as of January 2021, with topics ranging from “neurological” to “victories” to “resources and tips.” Despite this organization, there was an overflow of information that needed to be aggregated to be useful and to properly compare symptoms, pre-existing health conditions, and treatments. The members of the research team recognized that recording these experiences could be a powerful tool to highlight that this extended illness is not just happening to a select few COVID-19 cases but is happening to many across the world. We recognized that the number of people with Long COVID was only going to increase as the number of COVID-19 cases increased, and yet no one except patients seemed to be trying to figure out what was going on at the time.

Our research team came together in the Slack group shortly after the support group was formed, seeking answers to the questions we were asking of each other and of our doctors. Gina Assaf created the research group and launched the first survey on April 21, 2020, with the help of several other members. Shortly thereafter, the other core team members—Hannah Davis, Hannah Wei, Athena Akrami, and Lisa McCorkell—joined efforts to help analyze results and write the first report. All team members had relevant skills directly applicable to the work, and our diverse backgrounds blended together to create a multidisciplinary team that is hyphenate, female-led and spans 3 countries.

Our first survey was open from April 21 to May 2, 2020. We had a total of 640 respondents. We worked quickly to analyze the data and publish our first report because we understood how critical it was for this information to be public—not only did we want to work quickly to honor our respondents' time and energy but we also had a personal stake in it. Our first report was published on May 11—only 9 days after we began analyzing the data despite all of us working through debilitating symptoms.^38^

As we analyzed the survey, we paid attention to the questions the support group members had about their own data and the questions we were curious about ourselves. We recognized that a large portion of our respondents—47.8%—were unable to be tested and 27.5% tested negative despite exhibiting a similar symptom course. Instead of removing these valuable data from our survey, we used it. We found that the main difference between people who tested positive and people who tested negative was not necessarily the symptoms they experienced but how early in their illness they were able to be tested (day 10 of being symptomatic for those who tested positive vs day 16 for those who tested negative). This was further confirmed by our second survey study.^10^ We found that symptoms fluctuated and were not just respiratory but spanned many bodily systems, including neurological, cardiovascular, and gastrointestinal. We found that neurological symptoms were commonly reported by our respondents, despite being underreported in other COVID research. Importantly, the symptoms were not limited to fever, shortness of breath, and cough, indicating that restricting testing to only those experiencing all 3 of these symptoms missed out on a subset of patients with COVID-19.

### 3.2. Reception

Our report was the first research on Long COVID, released at a time when there was little discussion about the illness and what recovery of COVID-19 could actually look like. We posted our report on the Body Politic Support Group, and we received messages from support group members who found validation in seeing their experience reflected in a study of hundreds of other people. Many used the study to prove to their doctors that their symptoms are, in fact, real.

We paired our research with advocacy to better inform patients, doctors, medical organizations, and the general press, and our research was discussed in the first mainstream piece on Long COVID.^43^ Our research connected us with myalgic encephalomyelitis advocacy groups who have been experiencing similar symptoms but have been largely ignored by the research and medical community for decades. We presented our research to the World Health Organization,^39^ Centers for Disease Control and Prevention, and National Institutes of Health^8,39^ and were cited in the United Kingdom Parliament's first mention of Long COVID.^8,39,40^ We also contributed to the National Institute for Health and Care Excellence guideline on Long COVID.^25^ We have been cited in major medical journals such as the *British Medical Journal*, *JAMA*, and *Fatigue*; in publications such as *The Atlantic*, *MIT Tech Review*, and Cable News Network; and in the first guidelines put out for treatment of people with Long COVID.^29^

### 3.3. Additional research

Although we knew our first report captured important information, respondents who had not yet recovered had only been experiencing symptoms for an average of 40 days. As the months went by, our symptoms continued well beyond 40 days. Each of us experienced relapses and new symptoms, and those experiences were reflected in the support group. New information was being shared by members—antibody results, MRI results, and diagnoses. More and more people were joining our ranks as patients with Long COVID, and yet there were still few answers from the medical and research community on the science behind the illness and how best to treat it. Although the United Kingdom, Italy, and several other countries have started to follow-up with hospitalized patients with COVID-19 after they are released from hospital, little is known about the clinical course and return to baseline health for patients with outpatient illness and those with milder symptoms at the beginning that did not result in hospitalization.

Our second survey^9,10^ focuses on issues that many patients with Long COVID were discussing and sharing: antibody testing, the constellation of complex symptoms specifically neurological and cognitive, mental health and coping, diagnostics, and treatments. We made efforts to increase the diversity of our respondents by translating the survey into 9 languages and reaching out to communities hit hardest by COVID-19. From the resulting survey data, we characterized Long COVID's symptom profile (205 symptoms in 10 organ systems) and time course, as well as its impact on daily life, work, and return to baseline health, in an international cohort of 3,762 participants with confirmed or suspected Long COVID.^10^ All participants had illness onset between December 2019 and May 2020, allowing us to quantify symptom prevalence, time course, and severity over 7 months. Almost half of the respondents reported requiring a reduced work schedule compared with pre-illness and nearly a quarter were not able to work because of severity of their symptoms.

Our surveys are a reflection of our own experience and the experiences we read about in our support group. We thoughtfully chose each question, knowing that each one is critical. Our second survey took approximately an hour for respondents to complete—an amount of time that we know many patients with Long COVID would have difficulty completing in one sitting because of extreme fatigue. To account for this, we encouraged breaks at several points throughout the survey. We also email patients their responses if they request it so that they can keep this valuable information in their records to share with their doctors. Our partnerships with Long COVID community leaders in other countries, support groups, and Black, Indigenous, and people of color patient advocates helped to disseminate our survey, and we responded to feedback from participants regarding inclusive and accessible questions.

In our future work, we hope to partner with Black, Indigenous, and people of color community leaders in performing their own research, send follow-up surveys to track patients over time, and continue to advocate for patient-led research.

## 4. Gaps in care and research

Despite our progress, many gaps remain in research and patients' provision of care. Patients with Long COVID still need the following:

(1) Acknowledgement that this is an illness: Although the media and many public health professionals have acknowledged the existence of Long COVID, we are still experiencing disbelief from doctors and loved ones. We hear of (and personally experience) countless reports of doctors claiming patients' symptoms are caused by anxiety or are all in their head despite research documenting the existence of postviral illnesses.^23^ Many patients in our support group are being turned down for disability benefits and are not being believed by employers. Long COVID can be a debilitating illness, with many patients unable to work. Supportive policies must be in place to ensure that these patients are able to care for themselves and their families, and the recognition of Long COVID is the first step.
(2) An accurate estimate of the prevalence of Long COVID: Although there have been attempts at estimating the prevalence of Long COVID,^5,7,34^ most are flawed; one of the more publicized estimates assumes those with 4 or less symptoms as recovered, even if those symptoms are debilitating fatigue or cognitive issues.^30^ In a recent prevalence study by the UK's Office for National Statistics, respondents were not asked whether they experienced many commonly reported symptoms, including neurological and cognitive symptoms.^35^ Knowing how many patients Long COVID is affecting is critical for determining health care resources and estimating future impacts to employment. Moreover, the wider appreciation of the Long COVID prevalence and its implications may influence the public behavior around compliance to measures against the transmission of the virus. To estimate Long COVID properly, health organizations must develop, in partnership with patients, a standardized case definition of Long COVID. Then, contact tracers or researchers should follow-up with patients for several months after their isolation period ends to see if they meet the case definition. However, it must be noted that this may miss out on the portion of the population that is not tested or does not respond to contact tracers or researchers.
(3) Basic symptom management: The medical community must provide publicly available guidelines for basic symptom management, similar to what is provided in the *British Medical Journal*,^14,29^ by seeking input from patients with Long COVID and patients of other postviral illnesses. All symptoms that patients with Long COVID experience should be covered, particularly neurological symptoms that have so far largely been unaddressed. These guidelines must be accessible and in all languages. This will be especially useful for patients who may not have access to health care.
(4) Provision of care and research, being inclusive of all who show symptoms: As post-COVID clinics increase in number and more research is conducted on Long COVID, ensuring that all patients can receive care and are represented in research is critical. Currently, however, many clinics and research studies require a positive reverse transcription-polymerase chain reaction (RT-PCR) or antibody test to receive care and participate, which excludes a large proportion of patients with Long COVID.^3^ Many patients with Long COVID did not have access to testing, did not want to be tested, and/or may have not received an accurate test result, making it difficult to base inclusion criteria off of diagnostic testing. In diagnostic tests (RT-PCR or antigen), the likelihood of false negatives increases dramatically after day 3 of symptom onset.^19^ The site of sample collection can also play an important role in testing accuracy.^22,33^ Screening patients using antibody test results also does not reliably exclude previous severe acute respiratory syndrome coronavirus 2 (SARS-CoV-2) infection. Antibodies decline in the weeks after infection, in some cases falling below the threshold for seropositivity within only 2 months.^20^ The same study suggests that seroreversion occurs more often in patients who had a negative RT-PCR test result. It has also been reported that patients with neurological symptoms but minimal respiratory symptoms may fail to seroconvert.^12^ Although diagnostic and antibody testing do not capture every SARS-CoV-2 infection, symptoms can be a strong indicator of Long COVID. Several studies have demonstrated that, with the exception of change to smell and taste, symptoms are not significantly different between those who test positive for SARS-CoV-2 and those who test negative (or have not been tested) but who otherwise show strongly suggestive symptoms.^4,10,32^
Therefore, patients without a positive SARS-CoV-2 test result should not be ruled out of being considered patients with Long COVID. Medical professionals must consider the political, social, and physiological aspects of COVID-19 when providing care to prevent the unintentional construction of barriers. Researchers must also consider these issues when choosing participants and should work to have a deeper understanding of why some patients with Long COVID test positive and others do not despite having similar symptom courses.
(5) Aggressive research and investigation into the pathophysiology of symptoms: Since the beginning of the COVID-19 pandemic, hundreds of clinical trials have started to investigate how SARS-CoV-2 impacts the body. However, most of the focus has been on the acute illness. The pathophysiology of Long COVID, which may differ from that in the acute phase of COVID-19, warrants detailed investigation. The first step requires acknowledgement of the complexity and diversity of symptoms experienced by patients with Long COVID. Given the multiorgan impact of COVID-19,^42^ it is not clear whether the long-term sequelae will develop as unique complications or divergent pathophysiologies. Patients' insights about their phenotypic heterogeneity of symptoms should be incorporated into studies of pathogenesis and treatments.

Over a year into the COVID-19 pandemic, there is still more unknown than known. It is clear, however, that as patients, we are intimately aware of what we are experiencing. As the number of patients with Long COVID increases, it is critical for doctors, researchers, public health professionals, and policymakers to include patients in the conversation and support patient-led research. There is too much at stake not to.

## Disclosures

The authors have no conflicts of interest to declare.

This research did not receive any specific grant from funding agencies in the public, commercial, or not-for-profit sectors. All the activities were voluntary.
